# From the Editor-in-Chief

**DOI:** 10.14797/mdcvj.1114

**Published:** 2022-03-29

**Authors:** Miguel Quinones

**Affiliations:** 1 Houston Methodist DeBakey Heart & Vascular Center, Methodist J.C. Walter Jr Transplant Center, Houston Methodist, Houston, Texas, US

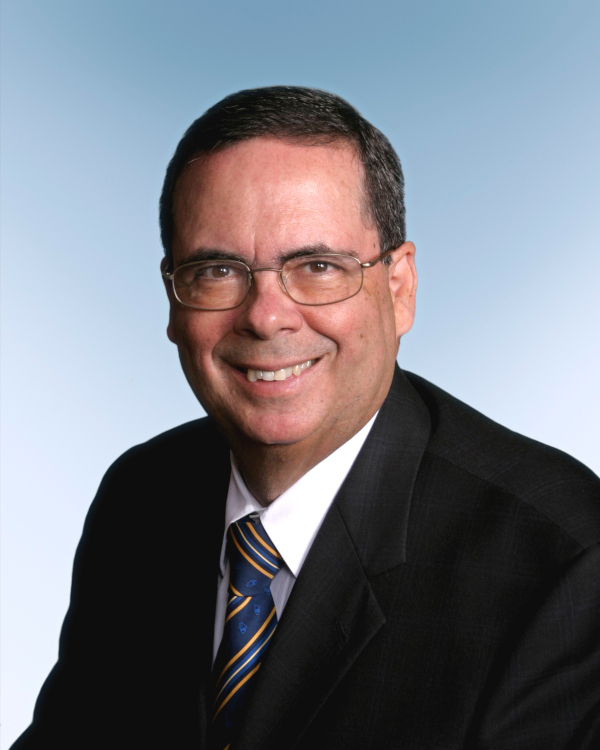



I am pleased to share this issue of the Methodist DeBakey Cardiovascular Journal. Though restrictive cardiomyopathies have previously posed seemingly unsurmountable challenges, it’s encouraging to see the promise of progress appearing on the horizon.

The review articles included here provide an insightful look at state-of-the-art practices and the newest advances for diagnosing and treating restrictive cardiomyopathies. In particular, their focus on amyloidosis and sarcoidosis exemplify how innovations in bioinformatics, imaging modalities, and drug development have impacted our ability to treat these diseases and to offer hope of future advances.

We invite you to share your experiences with cardiomyopathies and other challenging cases by submitting case reports and Multimodality Imaging manuscripts at the submission link on our website. We continually publish unusual, visually interesting, and informative cases in our online case reports issue 18.2, which is peer reviewed and indexed in PubMed. Find our year-round repository of case reports, interactive CME cases, and other articles with CME credit available in our Case Reports Collection.

As always, I appreciate your ongoing interest in the journal. I welcome your feedback as we strive to improve our publication in support of our educational mission of providing cardiologists and other physicians with the most current information about evidence-based practices and potential innovations.

Miguel A. Quiñones, MD, MACC, FASE

Editor-in-Chief

Methodist DeBakey Cardiovascular Journal

Winters Family Distinguished Centennial Chair

in Cardiovascular Education, Houston Methodist DeBakey Heart & Vascular Center

